# The Late Mr. James Lambert

**Published:** 1831-04-01

**Authors:** 


V. THE LATE MR. JAMES LAMBERT.

THIS gentleman, who, unfortunately for his own peace of mind, as well as that of many others, became involved in the disagreeable consequences of public criticism, paid the debt of nature at a very early period of life, and the disease under which he fell is remarkable in itself, and extremely unusual at his years. Mr. Lambert had always (at least for some years before his death) a very sickly appearance, which indicated some latent visceral affection. We have often met in the medical societies, and our impression always was, that he would die of phthisis. This, however, did not happen. It appears, from an unfinished memorandum in his own hand-writing, that in the month of May, 1830, he was affected with what he considered meningitis, which gave way to depletion, but left much debility. He tried change of air, and benefited from time to time; but still a pretty constant symptom was palpitation of the heart, on using any strong exercise. The loss of his father made a strong moral impression on his mind, and to palpitation was added dyspnœa, both which symptoms progressively increased till the day of his death.

*On dissection*, the right lung was found universally adherent to the costal pleura, and the greater portion of this lung was in a state of hepatization, in which were imbedded many depositions of a calcareous nature, some of them as large as a horse-bean. The heart was considerably enlarged. "Close to the heart, on slitting up the aorta, its internal surface was of a bright scarlet hue, as was the internal surface of the cardiac cavities, which were severally laid open, and, in doing so, a tumour became apparent, about the size of a large lime, occupying the septum between the two auricles, and, consequently, protruding into their cavities. It was soft, inelastic, and its outer surface covered by the lining membrane of the auricles, of a still deeper crimson colour than the rest of the cavity. On laying it open from the right auricle, it proved to be an aneurismal sac, communicating with the aorta by an opening large enough to admit the tip of the little finger, immediately behind the semilunar valves nearest to the right auricula propria, the two coronary arteries being apparently sound and opening into the aorta behind the two other semilunar valves. The mouth of the sac was of such a size, and in such a situation, as to be completely covered by the valve when in its perfect state of collapse, as during the contraction of the ventricle. The contents of the sac were about five drachms of coagulum, grumous in its centre, but becoming more dense as it approached the surface of the sac." The abdominal viscera, except the spleen, which was enlarged, appeared sound.—*Lancet, Jan.* 29.

Although we cannot quite coincide with our contemporary that Mr. Lambert died of a broken heart, yet we can well believe that anguish and perturbation of mind might have exasperated and accelerated a disease, the seeds of which must have been sown long before the unfortunate transaction took place which embittered this young gentleman's happiness. The calcareous depositions in the lungs—nay, the aneurism itself, were of considerable duration. Perturbation of mind is not a likely cause of such a very peculiar disease, though doubtless it would tend to aggravate any disease in existence at the time or previously. Often as we were opposed to Mr. Lambert in medical politics, we always considered him as a young man of superior talent; and had he lived, we have no doubt that he would have acquired reputation in his profession. His fate may teach us to avoid, as much as possible, those moral causes which agitate and embitter the little span of existence which is allotted to us on this transient scene of vanity and vexation of spirit!

